# Review of mosquitoes in Ireland and of relevant mosquito-borne pathogens

**DOI:** 10.1186/s13620-026-00331-9

**Published:** 2026-01-30

**Authors:** Annetta Zintl, Raysa Martins Hetherington, Anthony Murphy, Elsie Isiye, Angela Valcarcel Olmeda, Ciaran Lloyd, Maria Munita, Louise Mooney, Denise O’Meara

**Affiliations:** 1 UCD School of Veterinary Medicine, University College Dublin, Belfield, Ireland; 2 SETU School of Science and Computing, South East Technological University, Cork Road, Waterford, Ireland

**Keywords:** Mosquitoes, Mosquito-borne pathogens, Malaria, Yellow fever, West nile virus, Dirofilaria, Ireland

## Abstract

Throughout history, human societies have been greatly affected by mosquitoes and mosquito-borne pathogens such as malaria and yellow fever. On the other hand there have also been reciprocal effects as mosquito populations have been impacted by major societal changes such as agricultural intensification, urbanisation and international trade and travel, causing ever shifting patterns of disease exposure and epidemics. In contrast to continental Europe, mosquito-borne pathogens have not been a major concern in Ireland for many decades. However, the emergence of new invasive mosquito species and the changing epidemiology of mosquito-transmitted diseases in Europe calls for a re-evaluation of the species that occur here. This review provides an overview of the biology of mosquitoes, the species that occur in Ireland and exotic species that are currently expanding their geographical range in Europe. The most important pathogens transmitted by mosquitoes and their current status in Europe and Ireland are also discussed.

## Background

People are sometimes surprised to hear that there are mosquitoes in Ireland especially if they have the experience of being plagued by mosquitoes abroad. In fact there are at least 21 native mosquito species in Ireland, many of which are said to readily feed on humans [1]. However, anecdotally people rarely get bitten by mosquitoes in Ireland. The reason for this may be that one of the most notorious anthropophilic nuisance species, *Aedes vexans*, is apparently absent from Ireland. Its eggs which are deposited in soil subject to flooding and can survive for years, hatch simultaneously when water levels and temperatures rise. While always a nuisance, in bad years the resulting mass emergence can be such as to deter all leisure activity in the vicinity [2]. In contrast, there are only a few locations in Ireland where there are occasional reports of humans being bitten by mosquitoes. One of these is Drogheda in the north east of the country, where the responsible species is probably *Aedes detritus*, a common coastal species which breeds in estuaries and coastal marshes and readily feeds on humans. *Aedes detritus* is also the most important nuisance species in the UK [2].

The first species list for Irish mosquitoes was compiled by Ashe and colleagues [3], based on specimens collected by themselves and mosquitoes archived in the National Museum of Ireland and the British Museum. Historical records published by Irish entomologist Alexander Henry Haliday (1806–1870) and reports dating mostly from the early 20th century following the discovery of malaria were also included. As for mosquito-borne pathogens, *Plasmodium* spp, the causative agent of the most notorious mosquito-transmitted disease, malaria (also known as marsh fever or ague), historically caused occasional outbreaks in Ireland, as in the rest of Europe. In fact Oliver Cromwell and Queen Elizabeth I are supposed to have contracted the disease while in Ireland although McArthur (1951) argues that the ‘Irish ague’ that devastated Cromwell’s army in Ireland in 1649 was probably typhus not malaria [4]. Malaria also reportedly contributed significantly to the death toll of the ‘Eleven years war’ between 1641 and 1652 [5]. Another outbreak in Cork between 1854 and 1860 sparked by Irish soldiers returning from the Crimean War is well documented [6]. Over the second half of the 20th century malaria gradually disappeared from the continent and in 1975 Europe was declared malaria-free [2]. However, occasional autochthonous cases continue to be reported in Southern Europe and in the vicinity of large international airports (so-called ‘airport malaria’) [7–9]. To our knowledge the only locally acquired case of malaria reported in Ireland for many decades was a case of airport malaria in 2022. While competent vector species are native to Ireland and present on the island [1], there is no reservoir of infection which would serve to maintain the pathogen in the local mosquito population.

This review collates all currently available information on mosquitoes in Ireland and their biology. In addition invasive mosquito species and mosquito-borne pathogens that are of concern in continental Europe are discussed from an Irish perspective. The aim is to raise awareness of mosquitoes and mosquitoes-borne pathogens amongst public health officials and veterinary and medical professionals and provide basic information for potential future risk assessments and response plans.

### Biology of mosquitoes

The mosquito life cycle includes four larval stages all of which are aquatic and have specific environmental requirements. To ensure that these are met, female mosquitoes may spend several days searching for suitable breeding sites using visual (e.g. dark over light backgrounds) and chemical cues, such as odours from plants, organic material and bacterial breakdown products [10]. Semiochemicals released by conspecifics may also act as attractive oviposition pheromones or as deterrents by signalling high densities of conspecific competitors [11, 12]. In many species females lay their eggs singly or in batches directly on the water surface. These eggs do not enter dormancy or diapause and hatch within a day or two, once embryonal development is complete. In contrast others, especially those in the ‘floodwater’ and ‘snow-melt mosquito’ groups, lay their eggs into the moist soil or leaf litter just above the waterline, i.e. areas where water will return after rainfall or flooding. These eggs, which have varying degrees of resistance to desiccation and freezing, can remain dormant for months or even years [13]. Incidentally dormant eggs are the main route by which many invasive species colonise new areas. Water oxygen concentration and, to a lesser extent, temperature are the main cues that stimulate hatching of dormant eggs. For example in floodwater mosquitoes such as the nuisance mosquito *Ae. vexans*, hatching is triggered by declining oxygen levels as flooded pools heat up and become stagnant [13]. Immersion in deoxygenated water and/or addition of hatching agents which remove oxygen by chemical, physical, or biological means is also routinely used to stimulate egg hatching in colonies maintained under laboratory conditions [14]. In contrast hatching in many snow-melt mosquitoes is triggered by immersion in cold, oxygen-rich water [13]. All larval stages feed on microorganisms, algae, protozoa, invertebrates and detritus, by filter-feeding, scraping, shredding and predation. One of the reasons why mosquitoes only breed in stagnant, still or very slow flowing water is that larvae and pupae breath atmospheric air at the water surface using either a snorkel-like siphon (culicine larvae) or spiracular lobes on their dorsal surface (anopheline larvae). Species in the *Coquillettidia* genus obtain oxygen from aquatic plants by attaching themselves to the roots and stems of aquatic reeds and grasses and piercing their air-ducting tissues [13].

In many species, males emerge 1 to 2 days before females and as a result are generally somewhat smaller than females. They form mating swarms of up to a hundred individuals, typically around ‘markers’, prominent objects that contrast with the surrounding vegetation [2]. Females entering the swarm attract males via pheromones and the sound of their wing beat which has a lower frequency than that of males. Both the male and the female then match their wing-beat frequency to the flight tone of the other. This wing beat harmonisation is thought to synchronise their flight velocity facilitating mating on the wing [15]. Female mosquitoes mate only once in their lives, deriving sufficient sperm from a single copulation to fertilise all subsequent eggs [10].

Male mosquitoes feed exclusively on plant juices, sugars and floral nectar, and are able to utilise a range of plant material when flowers are scarce. Plant-based meals probably also represent an important food source for females especially before blood feeding and/or during gonotrophic development [11].

For the blood meal, females of different mosquito species have a preference for certain host species with anthropophilic species, preferably feeding on humans, ornithophilic species feeding on birds and zoophilic species feeding on other animals. Female mosquitoes may even have predilection sites on a host’s body (e.g. feet and ankles or the face) [11] and a considerable amount of research has focused on how female mosquitoes choose and locate their prey. Again, visual cues are important, even in nocturnal species which are more active during full moon, as mosquitoes can recognise contours, movement and light intensity, though whether they can also see colour is debated [10, 11, 13]. Other physical cues such as body heat and moisture are also detected. However, by far the most important stimuli are olfactory. The best known mosquito kairomone or attractant is CO_2_, which acts in a dose-dependent manner and enhances the effectiveness of other attractants typically present in vertebrate breath and sweat such as acetone, lactic acid, octenol, ammonia, carboxylic acids and urea, with their relative contributions indicative of host species [11, 12, 16]. Fatty acids produced by the skin microbiome are also strong stimulants and odours in specific locations such as the feet can guide mosquitoes to preferred feeding sites. In fact individual differences in skin and breath emanations are thought to explain why some individuals are apparently more attractive to mosquitoes and are bitten more frequently than others [11, 12, 16]. Females use their well-developed piercing and sucking mouth parts to first probe and then pierce capillaries in the skin to ingest blood 2 to 3 times their body weight [13]. They then find a place to rest and draw water out of the blood meal which is excreted as urine. Most species only feed outdoors (exophagous) although some readily enter buildings to feed (endophagous) and/or rest (endophilic).

### Mosquito species reported from Ireland

The species list shown in Table 1 has been compiled based on records published by Ashe et al. [3], Tuohy [17] and Schaffner et al. [1]. It includes 8 *Aedes* species (counting *Aedes geminus*, a sibling species of *Aedes cinereus*, separately), 4 *Anopheles* species (plus potentially 1 or 2 more species in the *Anopheles maculipennis* species complex), one *Coquillettidia* species, 2 *Culex* species and 5 *Culiseta* species. Species-specific information on preferred breeding and larval habitats and adult life style characteristics was collated from several sources [1, 2, 13, 18, 19].

**Table 1:** Mosquito species that have been reported from Ireland, preferred breeding sites and characteristics of adult life stages species records are based on the checklist compiled by Ashe et al. [3] and additional records published by Tuohy [17] and Schaffner et al. [1]

Species name	Preferred breeding sites and larval habitats	Adult female characteristics
*Aedes cantans* (*Ochlerotatus* *cantans*) [3]	Snow-melt/forest mosquito Open permanent/semipermanent meadow pools; shaded pools in deciduous or mixed forests with scarce aquatic vegetation but a thick layer of leaves at the bottom	Preferentially feeds on cattle and sheep, but also humans, horses, rabbits and birds; most active after sunset but in forests also during the day; frequently found in dense vegetation; fly short distances to open spaces such as pastures and river lowlands
*Aedes caspius* (*Ochlerotatus* *caspius*) [3]	Floodwater/coastal mosquito Halophilic; inland/coastal salt marshes and fens, rock pools; also in freshwater e.g. river valley floodplains	Readily bites humans and other mammals in urban and rural settings; females strongly exophagic but can enter houses and cattle sheds to feed; most active during dusk but also during the day and at night; highly resistant to heat and drought; can migrate for long distances (up to 10 km)
*Aedes cinereus* [3]	Floodwater/flood plain mosquito Tolerant to slightly acidic and oligotrophic conditions; temporary and semi-permanent waterbodies in floodplains and woodlands, sedge marshes, bogs (Sphagnum), sheltered edges of lakes	Feeds on mammals, including humans, mostly between dusk and dawn but also in shaded areas during the day; practically never met in open unshaded fields; can occur in masses and cause great annoyance to walkers; low migration range (< 800 m)
*Aedes detritus* s.l. (*Ochlerotatus detritus*) [3]	Floodwater/coastal mosquito Halophilic, tolerance to extreme salinity; semi-permanent brackish water bodies in estuaries and coastal marshes (*Salicornia*); stagnant drainage channels/lagoons with little aquatic vegetation; only occasionally in freshwater (possibly only in association with salt deposits); eggs can tolerate desiccation for > 1 year	Readily bites humans with potential to represent a serious nuisance; most common coastal species; predominantly active at dusk but also during the day; adults typically exophilic but can be seen to enter houses to feed; can migrate for long distances (estimated flight range up to 20 km)
*Aedes dorsalis* (*Ochlerotatus* *dorsalis*) [3]	Floodwater/coastal mosquito Halophilic; small, open permanent and temporary waterbodies in pastures, roadside, drainage ditches, preferably but not necessarily near saline waterbodies	Opportunistic feeder, attacks humans readily, painful bite; typically in open habitats e.g. pastures and mixed forests; exophilic but enters houses readily to feed
*Aedes geminus* [1]	Floodwater mosquito; sibling species of *Ae. cinereus* Preferred breeding sites resemble those of *Ae. cinereus*, often occur together in the same waterbody; somewhat less tolerant to acidic conditions than *Ae. cinereus*	Feeds on mammals, including humans
*Aedes punctor* (*Ochlerotatus* *punctor*) [3]	Snow-melt/forest mosquito Acidophilic (tolerant to pH < 4); temporary boggy water bodies with Sphagnum moss, swampy forests; larvae hatch at water temperatures just above 0 °C	Feeds on mammals including humans and birds, can represent significant local nuisance; adults prefer sheltered terrain, seldom migrate out of the forest
*Aedes rusticus* (*Ochlerotatus* rusticus) [3]	Snow-melt/forest mosquito Pools lined with dead leaves, ditches or depressions with *Carex*, *Phragmites* in swampy woodlands with a high level of groundwater, or, more rarely in flood plains; larvae can survive under a closed cover of ice	Readily feeds on humans but also other mammals and birds; adults prefer to stay in the shade in forested areas; do not migrate long distance (< 2 km)
*Anopheles algeriensis* [3]	Natural and artificial permanent well-shaded waterbodies e.g. ditches, canals, pools with rich vegetation (e.g. *Phragmites*), swamps and marshes; mostly freshwater, sometimes in brackish water (tolerance to slight salinity)	Feeds on humans and animals; exophilic, i.e. adults rest outside in dense vegetation and rarely enter houses/stables; not usually encountered near human habitation, populations typically of low abundance
*Anopheles claviger* [3]	Clean, permanent plant-rich waterbodies (*Lemna*), cool shaded habitats (e.g. pools with reeds sheltered by trees at the edge of ponds/lakes); also in artificial waterbodies such as wells, cisterns and water containers	Zoophilic, feeds on large domestic animals and humans; the first generation in a year may seek shelter in animal houses but later generations are exophilic and do not readily enter houses or stables; not usually encountered near human habitation; most widespread mosquito species in Britain
*Anopheles maculipennis* s.l. [3] (species complex comprising morphologically indistinguishable sibling species)	Standing or slow-moving (semi-) permanent plant-rich waterbodies, preferably with minimal vegetation and ample exposure to sunlight; also close to human settlements; Species reported in Ireland (but not confirmed): *An. maculipennis* s.s., *An. messeae*, *An. atroparvus* (as reviewed by Schaffner et al. [1])	*An. daciea*, *An. maculipennis* s.s. and *An. messeae*: predominantly zoophilic, but can bite humans *An. atroparvus*: anthropophilic
*Anopheles daciae* [1]	Member of the *An. maculipennis* complex	Zoophilic
*Anopheles plumbeus* [3]	Chiefly in water collected in tree holes of common deciduous trees (beech, elm, sycamore, ash, lime, oak), infused with tannins, wood pigment and salts and low in oxygen; also in rock holes and shaded ground depressions containing large amounts of organic matter; water catch basins, disused septic tanks; mostly in forests, rural areas, but also in gardens and parks	Feeds on mammals including humans, some populations show anthropophilic preference; most active during crepuscular period
*Coquillettidia richiardii* [3]	Permanent waterbodies, e.g. freshwater or slightly saline marshes, lakes, old river beds, estuaries, siltation zones (*Acorus*, *Typha*, *Phragmites*, *Glyceria*, *Sparganium*, *Ranunculus*, *Carex*)	Preferentially feeds on livestock, but also humans, birds and reptiles; frequent indoor feeding; bite after sunset, at night, and just after sunrise; usually stay near breeding sites, but can use ascendent air currents to invade areas of higher altitude
*Culex pipiens* [3] To our knowledge there are no records of biotype *Cx. pipiens molestus* to date	*Cx. pipiens pipiens*: ‘House mosquito’ Nearly any type of waterbody including ponds, pools, along river edges in still zones, inundation areas, tree holes, rock pools, flooded cellars, construction sites, water barrels, gutters, tires, tin cans, ornamental ponds and containers in gardens & church yards; in both heavy shade and full sun; can tolerate high levels of organic pollution and low levels of salinity; typical pioneer species (*Cx. pipiens molestus*: mostly restricted to underground habitats e.g. flooded cellars; can tolerate highly polluted water e.g. sewage storage and treatment plants)	*Cx. pipiens pipiens*: Ornithophilic (occasionally observed feeding on mammals, amphibians and reptiles); most active after dusk and before dawn; abundant in urban and suburban areas; short dispersal range (< 500 m); anautogenous (*Cx. pipiens molestus*: zoophilic, nuisance biter of people and large domestic animals; autogenous, i.e. able to develop the first egg batch without taking a blood meal)
*Culex torrentium* [17]	Same as *Cx. pipiens pipiens*: polluted and unpolluted natural and artificial waterbodies including tree holes, slow running streams, vegetation at the edges of lakes, semi-permanent pools, marshy areas, man-made ponds, reservoirs of sewage treatment plants	Strictly ornithophilic (never reported to bite humans); major role in enzootic virus transmission in bird populations
*Culiseta alaskaensis* [3]	‘Large snow melt mosquito’ Small permanent open pools with fallen leaves at the bottom and little aquatic vegetation, swamps	Feeds on mammals including humans
*Culiseta annulata* [3]	‘Large house mosquito’ Large range of permanent & semi-permanent, natural and artificial, shaded and open waterbodies, e.g. pools, ponds, ditches, troughs, rainwater barrels, gutters and drains; can tolerate high levels of organic pollution (manure water) and high salinity	Feeds on a broad range of mammals and birds; aggressive human biter and common nuisance species in the UK; active during the night; frequently enters houses and stables; most likely cause of mosquito bites acquired during the winter months; may be mistaken for invasive species due to its larger body size and distinctive leg stripes
*Culiseta litorea* [3]	Coastal species but not restricted to saltwater habitat; open, sunlit areas of semi-permanent pools, ponds and ditches with rich growth of vegetation (e.g. *Typha*); can tolerate slightly brackish water	Chiefly ornithophilic, occasionally feeds on reptiles and mammals, including humans
*Culiseta morsitans* [3]	Open and densely shaded areas of pools, small ponds and ditches rich in vegetation (*Cladium mariscus*), fens, reedbeds, slow moving water and vegetated margins of open water; can tolerate slightly brackish conditions	Chiefly ornithophilic, occasionally feeds on humans, reptiles and small mammals
*Culiseta subochrea* [3]	Preferably brackish but also freshwater habitats, including ditches, ponds, garden tanks	Feeds on domestic animals and humans; probably similar to *Cs. annulata* but more exophagic

Females of *Anopheles claviger*, the *Anopheles maculipennis* complex, *Coquillettidia richiardii*,* Culex pipiens*, *Culex torrentium* and *Culiseta annulata* lay their eggs, which hatch within a couple of days, directly on the water surface. In contrast the *Aedes* species, *Ae. vexans*, *Ae. caspius*, *Ae. cantans*, *Ae. punctor*, and *Ae. rusticus* and *Anopheles plumbeus* deposit their eggs in the damp soil or leaf litter at the edges of waterbodies where they can remain dormant for months or even years before hatching following immersion. As can be seen in Table 1, mosquito species differ considerably in their breeding site requirements and survival of the larvae is contingent on the correct choice of oviposition site. Larvae of opportunistic species such as *Cx. pipiens*, *Cx. torrentium* and *Cs. annulata* can develop in a wide range of waterbodies and containers, while others are much more specific with regard to water quality and waterbody type, in which case plant species can serve as potential indicators of their presence (e.g. sphagnum moss in the case of *Aedes punctor* or *Lemna* (duck weed) in the case of *An. claviger*). Some slow growing mosquito larvae prefer oligotrophic and slightly acidic conditions (e.g. *Ae. cinereus* and *Aedes geminus*), while others, especially coastal species, are halophilic and can tolerate low (e.g. *Anopheles algeriensis*, *Cq.* richiardii, *Cx. pipiens*, *Culiseta litorea*) or high levels of salinity (e.g. *Aedes detritus*, *Cs. annulata*). *Anopheles plumbeus* can use water-filled tree holes as oviposition habitats and has adapted to breeding in artificial containers such as water butts. This ability is shared by *Cx. pipiens* and *Cx. torrentium* larvae which can be found in diverse water-filled receptacles including discarded tin cans, tires and vases in graveyards. Many other species also readily breed in man-made structures such as irrigation/drainage channels, ornamental ponds, water features and cisterns (e.g. *An. claviger*, *Cx. torrentium*, *Culiseta subochrea*). The subspecies or biotype *Culex pipiens molestus* closely resembles *Cx. pipiens pipiens* genetically but expresses different behaviour [20]. In Northern temperate latitudes it exclusively breeds in subterranean habitats such as flooded mine shafts, basements and sewer tunnels. To our knowledge this species has not been recorded in Ireland to date.

Once the larvae have completed their development and pupated, the emerging adult mosquitoes are grouped into species that breed and rest close to their hosts and do not fly long distances (e.g. *Cx. pipiens*), species that disperse moderate distances (e.g. *Ae. rusticus*) and species that can migrate considerable distances (*Ae. caspius* and *Ae. detritus*). This dispersal behaviour is generally linked to flying ability with strong fliers readily entering open spaces, moderate flyers favouring woodlands or the edges of fields and rivers and weak flyers remaining near their breeding habitats. Moreover, females which feed preferentially on birds (e.g. *Cx. pipiens pipiens*) are most abundant at an altitude of 10 to 12 m, while zoophilic species which feed mainly on mammalian hosts (e.g. *Ae. cinereus*) are more frequently captured in traps placed closer to the ground. Most species are active between dusk and dawn, however many *Aedes* species (e.g. *Ae. cantans*, *Ae. caspius*, *Ae. detritus*) readily feed during the day, especially in shaded areas. All mosquitoes in the Irish species list are anautogenous, i.e. the females require a bloodmeal before they can lay their first batch of eggs. In the case of *Cx. pipiens molestus*, it is thought that food reserves accumulated by immature stages in the highly eutrophic larval habitats are sufficient to enable emerging females to complete development of her first egg batch without taking a blood meal.

### Invasive mosquito species in Europe

The importance of trade and travel for transporting mosquitoes has been recognised for centuries [21]. In fact, quarantine regulations for yellow fever from 1909 that specified that ships with yellow fever on board were not permitted to anchor less that 200 m from shore, was based on the known flight range of the vector, *Aedes aegypti* and designed to prevent infected mosquitoes from reaching land [22]. In recent decades this risk has significantly increased despite efforts to treat ships and aircraft for invasive mosquitoes and closely monitor ports and airports [21]. As already mentioned the most common route by which invasive species access new areas is via dormant eggs, often carried in used tires or hydroponic house plants such as ‘lucky bamboo’ [13]. Hence most invasive species that are currently of concern are *Aedes* species that readily breed in small artificial water containers and produce highly resistant eggs. Following invasion, their survival in new areas is often facilitated by increased ambient temperatures due to climate change and environmental degradation resulting in biodiversity loss and fewer natural competitors and predators. Moreover a reduction in other host species typically leads to increased human exposure [23]. Invasive synanthropic species, i.e. species that have adapted to living in close proximity to human settlements, also benefit from sprawling urbanisation and the availability of artificial and/or polluted aquatic habitats.

Currently the most widespread invasive species in Europe is *Aedes albopictus*, the ‘(Asian) tiger mosquito’, which according to the most recent ECDC distribution maps, is now established in Austria, Belgium, Bulgaria, Croatia, Cyprus, France, Germany, Greece, Hungary, Italy, Malta, Portugal, Romania, Slovakia, Slovenia, and Spain, and introduced into Czechia, Liechtenstein, the Netherlands, and Sweden [24]. Occasional incursions into the UK have also been reported [25]. Originally from Southeast Asia it is not associated with natural habitats such as wetlands, but prefers peridomestic environments [2]. The eggs are resistant to long periods of desiccation and freezing (−2 to −10 °C), the larvae highly competitive and able to survive in a variety of natural and artificial water receptacles including tree holes, water tanks, uncovered cisterns, empty cans, flower pots and discarded tires [18]. Adult female *Ae. albopictus* are also highly prolific and have a wide host range including mammals, birds, reptiles and amphibians. The species which is characterised by a black thorax with a single, silvery-white median line starting between the eyes and running the length of the dorsal side of the thorax, is a known vector of Yellow Fever virus (YFV), Dengue virus (DENV), Chikungunya virus (CHIKV), Zika virus (ZIKV) and *Dirofilaria immitis* [13]. In addition it is able to transmit avian *Plasmodium* spp however its epidemiological importance as a vector is debated [26]. *Ae. albopictus* has developed widespread resistance to common larvicides and adulticides [23].

*Aedes aegypti*, the ‘Yellow fever mosquito’, can also act as a vector for YFV, DENV, CHIKV and ZIKV [13]. Originally from tropical Africa it resembles *Ae. albopictus* in that its eggs are also resistant to desiccation and it has a strong preference for artificial breeding containers. However, in contrast to the tiger mosquito its eggs are not resistant to frost and its distribution in Eurasia which currently includes southern parts of Russia, Turkey, Georgia, Crimea and Egypt as well as points of introduction in the Netherlands [24], is limited by its inability to survive cold winters [23]. Morphologically it is characterised by silver scales in the shape of a lyre on the dorsal surface of the otherwise black thorax [18].

Two further invasive species from Asia, *Aedes japonicus* (‘Asian bush’ or ‘rock pool mosquito’) and *Aedes koreicus* also produce freeze- and desiccation resistant eggs [18] and introduced and established populations have been reported from several European countries [24]. None of the invasive species have been reported from Ireland to date.

### Mosquito-borne pathogens in Europe

Vector competence describes the ability of a mosquito species to acquire, maintain, proliferate and/or develop a pathogen to the infective stage [13]. This is distinct from vectorial capacity which is determined by the abundance of a mosquito species in a particular location and at a particular time, its biting behaviour and longevity. In other words even if a mosquito species is shown to successfully transmit a particular pathogens in the laboratory, this does not necessarily mean that it is an important vector unless it is present in large enough numbers and at the right time to feed on and infect susceptible hosts. On the other hand mosquito abundance and seasonality are both parameters that are directly affected by man-made disruptions of climate and habitats. It is also important to point out that the estimated mosquito lifespan of 30 days that is often used in transmission models may not apply to mosquitoes living in temperate areas. For example *Ae. cantans* females have been reported to survive for over 60 days in the UK while hibernating populations of female *Culex pipiens* overwinter from July/August to the following April/May [27].

For a mosquito-borne pathogen to become established and persist in a host population it must be able to infect large enough numbers of hosts and persist in their tissues long enough for naïve mosquitoes to become infected when feeding on them. This can be challenging particularly during the winter months in temperate locations as most mosquito species overwinter as eggs or larvae. These will only turn into infective adults if the pathogen is transmitted vertically. Even in species that overwinter as mated females (e.g. *An. maculipennis* sensu lato, *Cx. pipiens*, *Cs. annulata*), the females typically only feed on plant sugar before becoming inactive for the winter, i.e. they do not take a blood meal before going into diapause [21]. Again for these females to be infective at their first feed in spring the pathogen has to be able to be passed vertically, otherwise they must feed on an infective host first.

Historically the most significant mosquito-borne disease in Europe was malaria caused by the protozoan parasites, *Plasmodium falciparum*, the cause of malignant tertian malaria, *Plasmodium vivax* and *Plasmodium ovale*, both of which cause benign tertian malaria, *Plasmodium malariae* (quartan malaria) and *Plasmodium knowlesi* [13]. All of these species are solely transmitted by anopheline mosquitoes. Following ingestion by the mosquito they undergo sexual reproduction in the midgut, resulting in the production of sporozoites which invade the salivary glands from where they are injected during the next blood meal. The time that it takes to complete this development in the mosquito, known as extrinsic incubation period, is directly affected by ambient temperatures. If it exceeds the feeding intervals of the mosquito, successful transmission is unlikely [19]. It is thought that the main *Plasmodium* species that was endemic in Europe, including Ireland, was *P. vivax*, as it can complete its extrinsic development at relatively low ambient temperatures, in areas with a summer isotherm of 16 °C, while *P. falciparum* requires a summer isotherm of 20 °C limiting it to the South of Europe [13]. Generally, malaria transmission in northern and western Europe was probably always somewhat precarious, causing discontinuous epidemics with annual maxima. During the 1950’s and 60’s malaria gradually disappeared from the European continent largely as a result of widespread drainage and canalisation, changed agricultural practices, improved socio-economic conditions and sanitation [2]. Under the European Disease Surveillance System, coordinated by the European Centre for Disease control and Prevention, between 5,000 and 7,000 malaria cases are reported in the EU/EEA area annually (0.9 to 1 cases per 100,000 population), the vast majority of which (over 99%) are travel-related [9]. Of the small number of locally-acquired infections most are considered airport-related, resulting from exposure to infected mosquitoes that have travelled in the hold. In addition there are sporadic reports of autochthonous cases from some rural areas in France, Greece, Italy and Spain [7–9, 13]. Importantly the species that were responsible for the transmission of malaria in Europe in the past, *An. maculipennis* s.l. complex (*An. atroparvus* and to a lesser extent, *An. messeae* were historic malaria vectors in Britain [2]) as well as, potentially, *An. claviger*, *An. algeriensis* and *An. plumbeus*, are still widespread throughout the continent and are present in Ireland [1]. However, in the absence of significant numbers of infected people serving as a reservoir of infection, it is unlikely that the disease will become reestablished in Europe.

While there is a long list of mosquito-transmitted viruses, the most important ones in the European context are, for historical reasons, YFV, DENV, CHIKV, ZIKV and WNV. Yellow Fever Virus, which is chiefly a pathogen of the Americas, was frequently imported into Europe by returning sailors resulting in a number of well-documented outbreaks throughout the 19th century, particularly in Portugal and Spain, but also France, Italy and the UK [28]. A classic example was the so-called ‘plague of Barcelona’ in 1821 which started in June with the arrival of several merchant ships that had travelled in convoy from Havana and Vera Cruz with a number of infected people on board. Many friends and family members who boarded the ships to welcome the returning sailors, as well as numerous people working in the ports, died suddenly from an unidentified disease. The port was eventually placed under quarantine but not before the disease had spread throughout Barcelona and neighbouring cities in Catalonia, as well as the Balearics and Marseilles (via a Danish ship with sick people on board). As temperatures started to decline in autumn and mosquitoes became less active, cases numbers began to fall and by the middle of December 1821 the outbreak was officially declared over. By that time the epidemic is estimated to have claimed the lives of a sixth of the population of Barcelona [28]. The virus was isolated in 1927 and vaccines based on two virus strains were developed in the 1930 s, one of which is still in use today. Yellow fever is no longer a health threat in Europe although it continues to be a major health concern in Latin America and Africa.

The main vector for yellow fever in Europe was *Ae. aegypti*. At that time it was endemic in Southern Europe with well-established populations throughout the Mediterranean Basin including France, Greece, Italy, Portugal and Spain. During the 1950’s and 1960’s the mosquito disappeared from continental Europe, probably as a result of changed agricultural practices, land drainage and the introduction of piped water to rural villages [29]. Vector control programmes, though implemented primarily to control malaria, were likely also a factor. However the mosquito is still present in neighbouring countries and is occasionally re-introduced causing some experts to warn that it could become reestablished in Europe [30].

*Aedes aegypti* is also the primary vector of DENV, CHIKV and ZIKV. In addition the three viruses can be transmitted by the tiger mosquito, *Ae. albopictus*. As mentioned earlier, this highly adaptable tropical mosquito species has developed tolerance to lower ambient temperatures and is now endemic in much of Southern and central Europe [24]. Neither Dengue, nor Chikungunya or Zika are endemic to Europe but they are frequently imported by the travelling public [30] and in areas where *Ae. albopictus* is established, these introductions can give rise to local transmissions. In 2025 locally acquired cases of CHIKV amounted to 776 in France and 374 in Italy [31]. However, temperatures in Europe do not support long-term virus transmission (e.g. optimal DENV-transmission occurs at 31 °C, CHIKV transmission at 24 to 26 °C, and ZIKV transmission at 29 °C [32–34]). As a result, locally transmitted outbreaks typically run their course over the space of a couple of months and disappear altogether once temperatures decrease in autumn and winter.

The same used to be true for WNV. Until 1999 the virus was largely limited to Africa from where it was occasionally introduced into Europe, mostly by migratory birds, causing sporadic outbreaks in the human population especially in the Mediterranean region, Romania and Russia which did not persist during the winter months [35, 36]. Since then the situation has changed as the virus seems to have acquired the ability to overwinter in Europe, chiefly by persisting, for several months, in the tissues of infected birds (especially passerines). Moreover the virus can be transmitted vertically in the eggs of the female mosquito. The main vectors of WNV are *Cx. pipiens pipiens*, *Cx. pipiens molestus*, *Cx. torrentium* and *Cx. modestus* all of which are endemic and present in large numbers throughout Europe. *Cx. pipiens pipiens* and *Cx. torrentium* are largely ornithophilic and play an important role in the transmission of WNV within bird populations, while the anthropophilic *Cx. pipiens molestus* may contribute to human WNV exposure in suburban and rural environments. However, *Cx. modestus* and hybrids produced by the two subspecies, *Cx. pipiens pipiens* and *Cx. pipiens molestus*, are thought to act as the main bridge vector between avian and mammalian populations by feeding on a variety of bird and mammal species, including humans [37, 38]. Overwintering of the virus may also be helped by the fact that all life stages of the underground-dweller, *Cx. pipiens molestus*, occur throughout the year (even in countries as far north as the UK) [36].

In contrast to DENV, CHIKV and ZIKV, most human cases of WNV infection reported to ECDC are locally acquired [39]. Because this virus is a major pathogen of birds, especially passerines, and also affects horses, WNV outbreaks are frequently associated to high crow mortality and an increase of equine encephalitis cases [36]. In 2023, there were 728 reported human cases in Europe, compared to 153 outbreaks in equids and 251 in birds [39]. That year WNV was also detected for the first time in the UK, in two pools of female *Aedes vexans* mosquitoes collected in Nottinghamshire, England [40]. However the authors found no evidence of active transmission of WNV in the UK and suggested that current climatic conditions would not sustain virus circulation (virus transmission is optimal at 23 to 26 °C [41] with some variability between strains, and historical strains requiring lower temperatures than more recent ones [42]). Moreover *Ae. vexans*, though a competent vector for WNV, is an uncommon species in the UK [40], and there is limited exposure of humans to the known bridge vector species, although there may be a potential risk to horses [37] Currently there are three vaccines for use in horses that have been granted authorisation by the European Medicines Agency and can be used under license in Ireland [43].

Even more important than WNV, from a veterinary point of view, are the mosquito-borne filarial heartworms, *Dirofilaria immitis* and *Dirofilaria repens*. Primarily pathogens of dogs and wild canids, the worms can also infect humans and cats. Both are transmitted by *Cx. pipiens* s.l. in addition to several other mosquito species, including *Ae. vexans*, *Ae. caspius* and potentially, *Ae. albopictus* [27, 44]. Following ingestion of microfilariae with the blood of an infected host, the parasites moult twice in the mosquito’s Malpighian tubules. The resulting infective stage 3 larvae migrate to the mouthparts and invade a new host during the next blood meal. As with all vector-borne pathogens this extrinsic development is highly temperature-dependent, requiring 8 to 9 days at 30 °C, 10 to14 days at 26 °C, 17 days at 22 °C, and 29 days at 18 °C [27]. It has been argued that increasing environmental temperatures due to climate change have facilitated the expansion of both heartworm species on the European continent, becoming endemic in areas which were considered *Dirofilaria*-free only one or two decades ago [45]. Regarding transmission of *Dirofilaria* in the UK, Medlock and colleagues concluded that even if indigenous transmission were to occur it would likely be localized and restricted to brief periods during warmer years, although they conceded that these could expand and become more common as a result of climate change [27]. They also stressed the fact that autochthonous transmission depended on the availability of infected dogs. While there has been a significant increase in the number of dogs coming from endemic areas into the UK since the introduction of the Pet travel scheme in the early 2000’s, reports of imported cases have been limited [44]. In Ireland, the first case of *D. immitis* was reported in 2020 in a dog referred to the Veterinary Hospital in University College Dublin. The animal had been imported from the Canary Islands following rehoming from an animal shelter [46]. Over the last two years just one case of heartworm infection was diagnosed in the UCD Veterinary Hospital. Nevertheless continued vigilance among veterinary practitioners in the UK and Ireland is important to ensure infections do not go undetected or underreported [44].

### Monitoring and control of mosquito populations

Numerous methods have been developed for the collection and monitoring of mosquitoes including traps for flying or resting adults, collection methods for larvae and oviposition traps for eggs [47]. Some of these are specific for certain species (e.g. traps set at specific times in specific sites, or baited with particular scents) while others can be used to collect both male and female mosquitoes of a broad range of species. Flying adults are usually collected using traps that employ CO_2_ and other attractants such as lactic acid, 1-Octen-3-ol, sweetscent and/or light and a suction fan that directs the mosquitoes into the capture net. Resting adult mosquitoes can be caught using aspirators or hand nets while larvae are collected in their various breeding sites by netting, dipping or aspirating. While any water container can act as an oviposition trap (or ovitrap), using a black container with a wooden stick or piece of polystyrene as oviposition support and an oak leaf or hay infusion can enhance the attractiveness of the trap. Ovitraps are often used to monitor invasive mosquito species as most of them are container breeders.

The most cost-effective and least environmentally detrimental control measures for mosquitoes include covering or removing artificial breeding sites [19] and applying spores of a highly specific larvicidal bacterium, *Bacillus thuringiensis israelensis* (Bti), to larger, natural sites. Once consumed by mosquito larvae, Bti spores release toxins into the gut, causing the larvae to stop eating and die [48]. Bti is non-toxic to humans, mammals, birds, fish, plants and most aquatic organisms. In fact, the only other organisms directly affected by Bti are non-biting midges and blackflies. However, by effectively removing mosquito larvae from a habitat, Bti can have a severely detrimental impact on the food web causing a drastic reduction in natural predators such as dragonflies, spiders and insectivorous birds [49]. Botanical repellents such as citronella, pyrethrum or neem oil and DEET-based products are frequently used as personal protection against blood feeding females, although there are safety concerns about the latter, especially when used on children [13, 19]. Other measures, especially against anthropophilic endophilic species, include window and door screens and bed nets and curtains treated with conventional or long-lasting insecticides [13].

## Conclusions

To our knowledge there are currently no exotic mosquito species in Ireland and while travel-related cases are regularly reported [50], there is no evidence so far of any locally acquired mosquito-borne diseases. However, climate change together with increased international travel and trade has facilitated the sustained ingress and spread of invasive species such as *Ae. albopictus* and pathogens such as WNV across Europe and it is likely that, in time, Ireland will also be affected. Ongoing surveillance to ensure early detection of invasive mosquito species and mosquito-borne pathogens is crucial for the timely implementation of effective control measures. Reducing environmental degradation will also help to lessen the impact of any such incursions, which are frequently favoured by natural homogenisation and biodiversity loss [23]. Protecting natural ecosystems will also help to boost populations of natural predators and competitors including native mosquito species which are generally inconspicuous, causing many people to be surprised when they hear that there are, in fact, mosquitoes in Ireland.

## Data Availability

No datasets were generated or analysed during the current study.

## Abbreviations

Bti: Bacillus thuringiensis israelensis
CHIKV: Chikungunya virus
DENV: Dengue virus
ECDC: European Centre for Disease Prevention and Control
WNV: West Nile Virus
YFV: Yellow Fever virus
ZIKV: Zika virus
